# Mucosa-Associated Microbial Profile Is Altered in Small Intestinal Bacterial Overgrowth

**DOI:** 10.3389/fmicb.2021.710940

**Published:** 2021-07-30

**Authors:** Jia Li, Ru Zhang, Jinxia Ma, Shuai Tang, Yuan Li, Yi Li, Jun Wan

**Affiliations:** ^1^Medical School of Chinese PLA, Beijing, China; ^2^Department of Gastroenterology, The Second Medical Center & National Clinical Research Center for Geriatric Diseases, Chinese PLA General Hospital, Beijing, China; ^3^Department of Gastroenterology, The 983th Hospital of Joint Logistic Support Force of PLA, Tianjin, China

**Keywords:** SIBO, microbiota, mucosa, duodenum, ileum, 16S rRNA gene sequencing, biomarker

## Abstract

The overall gut microbial profile of patients with small intestinal bacterial overgrowth (SIBO) has not been thoroughly investigated. We investigated the microbial communities of mucosal specimens from the duodenum, ileum, sigmoid colon, and feces of patients with and without SIBO, as diagnosed by lactulose breath testing. The bacteria present in the mucosal and fecal samples were identified using 16S rRNA gene sequencing. Further analysis was performed using the linear discriminant analysis (LDA) effect size method, random forest analysis, and receiver operating characteristic analysis. The microbial diversities of the fecal samples were significantly lower than those of the mucosal samples from the duodenum, ileum, and sigmoid colon (*P* < 0.001, *P* < 0.001, and *P* < 0.001, respectively), while the bacterial compositions of the ileac mucosal samples and sigmoid mucosal samples were similar. The bacterial composition of either the fecal or duodenal mucosal samples were significantly different from those of the other three groups (ANOSIM *R* = 0.305, *P* = 0.001). The bacterial compositions of the mucosal samples of the duodenum, ileum, and sigmoid colon in the SIBO + subjects were significantly different from those of the SIBO− subjects (ANOSIM *P* = 0.039, 0.002, and 0.007, respectively). The relative abundances of 7, 18, and 8 genera were significantly different (LDA score > 3) in the mucosal samples of the duodenum, ileum, and sigmoid colon between the SIBO + and SIBO− groups. Four genera (Lactobacillus, Prevotella_1, Dialister, and norank_f__Ruminococcaceae) showed similar changes among the mucosal samples of the duodenum, ileum, and sigmoid colon in the SIBO + subjects. A signature consisting of four genera in the duodenal mucosa, three genera in the ileac mucosa, or six genera in the mucosa of the sigmoid colon exhibited predictive power for SIBO (area under the curve = 0.9, 0.93, and 0.87, respectively). This study provides a comprehensive profile of the gut microbiota in patients with SIBO. Dysbiosis was observed in the mucosa-associated gut microbiome but not in the fecal microbiome of patients with SIBO. Furthermore, we identified mucosa-associated taxa that may be potential biomarkers or therapeutic targets of SIBO. Further investigation is needed on their mechanisms and roles in SIBO.

## Introduction

Small intestinal bacterial overgrowth (SIBO) is defined as having excessive amounts and/or abdominal bacterial species in the small intestine (Ghosh and Jesudian, 2019). This disease has been implicated in a series of gastrointestinal and non-gastrointestinal symptoms and is thought to be linked to a growing number of disorders (Lee et al., 2019; Shah et al., 2020; Tremellen and Pearce, 2020; Song et al., 2021). The microbial community in the human digestive tract is a complex ecosystem that is vital to health but also a potential driver of various diseases (Bäckhed et al., 2012; Ringel et al., 2015). To date, the gut microbiota composition in patients with SIBO has yet to be established.

The culture of jejunal aspirates has long been considered the gold standard for the diagnosis of SIBO (Erdogan et al., 2015). However, its clinical application is limited due to its invasive nature and potential for false positives due to contamination (Quigley et al., 2020). In addition, since several species of bacteria cannot be cultured (Eckburg et al., 2005), bacterial culture results do not reflect the real spectrum of the gut microbiome. Lactulose or glucose hydrogen and methane breath testing is currently the most widely used non-invasive technique for the diagnosis of SIBO (Quigley et al., 2020). In recent years, the introduction of 16S rRNA sequencing has greatly expanded our ability to characterize the gut microbiome and increased our understanding of previously unculturable microbes (Vaughan et al., 2000).

To date, molecular studies have yet to characterize the intestinal flora in SIBO. While several studies have described the intestinal microbiome and its association with human disease, few have characterized the gastrointestinal mucosal flora. Additionally, the majority of studies on dysbiosis have only been conducted on feces as they are easily collected (Jackson et al., 2018; Zhuang et al., 2018; Lema et al., 2020). However, fecal bacteria are not necessarily representative of the local gastrointestinal flora (Zoetendal et al., 2002; Willing et al., 2010) that directly interact with the host. Thus, the mucosa-associated microbiota in humans, especially in the distal small intestine, have not been comprehensively investigated due to sampling challenges (Kastl et al., 2020). It is also widely accepted that SIBO is caused by retrograde shifts of bacteria in the large intestine (Korterink et al., 2015). Furthermore, the microbial compositions of the proximal and distal intestine have previously been found to be different (Yang et al., 2020), with the distal intestinal microbiota potentially being more susceptible to invasion by colorectal bacteria in cases of SIBO.

In this study, we investigated and compared the spectra of mucosa-associated microbiota in the upper (duodenum), middle (ileum), and lower (sigmoid colon) gastrointestinal tract as well as fecal microbiota using 16S rRNA gene sequencing in patients with and without SIBO, as diagnosed using lactulose breath testing. A combination of fecal and mucosal studies was also performed to reveal a comprehensive profile of the microbiome in patients with SIBO. In addition, we identified a microbial signature that could predict the presence of SIBO in suspected patients.

## Materials and Methods

### Study Cohort

Patients aged 18–75 years with unexplained intestinal gas, bloating, diarrhea, abdominal pain, and functional dyspepsia that were suspected to be due to SIBO were prospectively recruited. Exclusion criteria included the following: a history of active inflammation, microscopic colitis, inflammatory bowel disease, food allergies, cancer, immune diseases, and severe systemic, cardiac, renal, hepatic, metabolic, and psychiatric diseases; intake of antibiotics or proton-pump inhibitors (PPI) in the last 4 weeks; intake of promotility agents, laxatives, or probiotics in the past week; current pregnancy; inability to endure endoscopy; or inability to obtain informed consent.

Demographic information, height, weight, and previous medical history of abdominal surgery were obtained at the time of enrollment. Clinical symptoms were evaluated using the gastrointestinal symptom rating scale (GSRS) (Svedlund et al., 1988) consisting of 15 items, including abdominal distension, heartburn, acid regurgitation, abdominal pain, sucking sensations in the epigastrium, nausea and vomiting, borborygmus, eructation, increased flatus, decreased passage of stools, increased passage of stools, loose stools, hard stools, urgent need for defecation, and feeling of incomplete evacuation. Each item was evaluated using a scale of 0 to 3, with 0 showing no symptoms, and 3 showing extremely severe symptoms. The baseline information of each patient was collected by the same nurse prior to the evaluation of SIBO to minimize bias.

### Lactulose Hydrogen and Methane Breath Testing

All participants underwent lactulose hydrogen and methane breath testing (LBT) for diagnosis of SIBO and classified as either SIBO + or SIBO−. The participants were instructed not to eat fermentable foods and alcohol on the day prior to the test. They were also asked to fast for at least 12 h before the breath test, avoid smoking on the day of the test, and avoid exercise during the breath test. The participants cleaned their teeth on the morning of the breath test and gargled prior to testing to reduce fermentation of lactulose by oral bacteria. After the baseline expiratory samples were collected, the participants were instructed to take 10 g of lactulose orally (Duphalac, Abbott). Breath samples were then collected every 15 min until 90 min for a total of seven times. Hydrogen and methane concentrations were detected using a Quintron BreathTracker (Quintron Instrument Company, Milwaukee, WI, United States). A positive breath test standard was determined as follows (Rezaie et al., 2017; Pimentel et al., 2020): hydrogen rise above baseline ≥ 20 ppm by 90 min or methane ≥ 10 ppm at any point during the test within the 90 min.

### Sample Collection

Fresh fecal samples were collected before or at the time of gastroscopy in the hospital. The participants were instructed to remove the outer layer of their feces with a sampling spoon and collect the central portion into a sterile tube. Gastroscopy was performed to obtain one sample of the distal duodenum (5–10 cm below the major duodenal papilla) for biopsy as previously described (Li et al., 2018). The following day, colonoscopy was performed after bowel preparation with the oral compound polyethylene glycol electrolyte powder. One mucosal sample of the sigmoid colon (25–30 cm from the anus) was obtained during the insertion to prevent contamination by bacteria in the proximal colon. The enteroscope was then advanced past the ileocecal valve to obtain one sample of the distal ileum (5–10 cm from the ileocecal valve) for biopsy. All endoscopic procedures were performed by the same senior gastroenterologist. Each sample was placed in a sterile tube and immediately stored at −80°C for subsequent 16S rRNA sequencing analysis.

### DNA Extraction and 16S rRNA Gene Sequencing

Fecal DNA was extracted using the E.Z.N.A. Soil DNA Kit (Omega Bio-tek, Norcross, GA, United States), and mucosal DNA was extracted using the FastDNA Spin Kit for Soil (MP Biomedicals) according to the manufacturers’ instructions. The concentrations and purities of the extracted DNA were measured using a NanoDrop2000 UV-vis spectrophotometer (Thermo Scientific, Wilmington, United States), and the qualities of the extracted DNA were evaluated using 1% agarose gel electrophoresis. The V3-V4 regions of the bacterial 16S rRNA gene were amplified using primers 338F (5′-ACTCCTACGGGAGGCAGCAG-3′) and 806R (5′-GGACTACHVGGGTWTCTAAT-3′). Sequencing was performed using a MiSeq PE300 platform (Illumina, San Diego, CA, United States) at Shanghai Majorbio Bio-pharm Technology Co., Ltd.

Raw sequencing data were qualified and stitched using the Trimmomatic and FLASH software, respectively. Briefly, low-quality reads (those with nucleic acid lengths less than 50 bp after quality control, with quality values less than 20, or containing N bases) were filtered. Based on the overlapping relationships between the paired-end (PE) reads, they were merged into sequences with minimum overlapping lengths of 10 bp. The maximum error ratio of the overlapping area of the spliced sequences was set at 0.2, and sequences that did not meet this requirement were removed. The samples were distinguished according to the barcodes and primers at both ends of the sequences, with the directions of the sequence being adjusted. The data obtained were then optimized. The resulting sequences were processed using the QIIME software (version 1.9.1). All sequences with 97% similarities were clustered using the URARese software (version 7.1), and chimeras were removed in the clustering process to obtain sequences representative of OTUs. Lastly, taxonomic analysis of the OTUs was performed using the RDF Classifier software (version 2.11) and compared with the Silva database (SSU132) at a comparison threshold of 70%.

### Bioinformatics and Statistical Analysis

The richness and diversity of the microbial community were compared using alpha-diversity analysis, which considers diversity indices (Shannon and Simpson), richness indices (Sobs, Chao, and Ace), and sequencing depth indices (coverage index), using mothur software (version v.1.30.1). Alpha-diversities between groups were analyzed using the Wilcoxon rank-sum test. Beta-diversities were used to compare the differences in species compositions between groups analyzed using PCoA and PCA analysis based on binary_jaccard, abund_jaccard, and bray_curtis distance subjected to ANOSIM analysis. The differences in species abundance of the microbial community among several groups were evaluated using the Kruskal–Wallis rank-sun test. The differences in species abundance of the microbial community between groups and the effects of each differentially abundant taxon were assessed using the Wilcoxon rank-sum test or the linear discriminant analysis (LDA) effect size (LEfSe) method (Zhuang et al., 2018; Liu et al., 2019), which emphasized statistical significance and biological correlation. The threshold was set at LDA level > 3. Random forest analysis, a machine learning procedure, was used to identify taxa to build prediction models to distinguish between the SIBO + and SIBO− subjects, area under a receiver operating characteristic (ROC) curve (AUC) was used to assess the performance of random forest prediction model. Specifically, random forest analysis was conducted to rank the importance of species, and AUC scores were calculated for the prediction model with the top N species in importance. In the coordinate axis of validation figure, X represented the number of genera in the importance ranking and Y represented the AUC value. In building the prediction model, the corresponding X value of the solid point with the highest Y value was considered the ultimate number of top species. The performance of the prediction model was evaluated for its discriminative power using ROC curve. All sequencing data were analyzed using the R software (version 3.3.1); randomForest package and plotROC package were the machine learning packages used.

In addition to the sequencing data, other data were statistically analyzed using the IBM SPSS Statistics software (version 25.0). For continuous data analysis, the Mann–Whitney *U*-test was used. For categorical data analysis, Fisher’s exact test was used. In all test results, the differences were considered statistically significant when the double-tailed *P* value was < 0.05.

### Study Size

The sample size of the subjects was determined based on the number of cases available during the study period. No formal sample size calculations were conducted prior to the commencement of this pilot study.

### Ethical Considerations

The study protocol was approved by the Institutional Ethical Review Committee of the Chinese PLA General Hospital (approval reference number: S2018-081-02). All participants provided written informed consent.

### Data Accessibility

The sequencing data is available in National Center for Biotechnology Information under the Sequence Read Archive (SRA) with the BioProject No. PRJNA728515.

## Results

### Demographic and Clinical Characteristics of SIBO + and SIBO− Subjects

A total of 34 subjects were recruited, of which three were excluded from the analysis due to lack of information or factors related to sample handling, storage, or experimental operation. A total 31 subjects (19 men and 12 women) were eventually included. In some subjects, samples from all four sites were not collected. A total of 25 fecal, 24 duodenal, 26 terminal ileal, and 25 sigmoid colonic samples were collected.

According to the LBT, 17 subjects were classified into the SIBO + (10/17 male) group, and 14 subjects were classified into the SIBO− (9/14 male) group. There were no significant differences in sex distribution, age, BMI, or history of abdominal surgeries. The scores of abdominal distension and feeling of incomplete evacuation were higher in the SIBO + group than in the SIBO− group (*P* = 0.002 and *P* = 0.012, respectively). There were no differences in the scores of abdominal pain, heartburn, acid regurgitation, sucking sensations in the epigastrium, nausea and vomiting, borborygmus, eructation, increased flatus, decreased passage of stools, increased passage of stools, loose stools, hard stools, and urgent need for defecation, and total scores between the SIBO + and SIBO− groups. The demographic and clinical characteristics of all subjects are displayed in Table 1.

**TABLE 1:** Demographic and clinical characteristics of SIBO + and SIBO− subjects.

	SIBO + subjects (*n* = 17)	SIBO− subjects (*n* = 14)	*p* ^a^
Gender, male, and *n* (%)	10 (58.8%)	9 (64.3%)	1.000
Age, years, and median (IQR)	62 (10)	59 (8)	0.905
BMI, median (IQR)	23.2 (4.8)	24.3 (3.0)	0.197
History of abdominal operation, *n* (%)	3 (17.6%)	1 (7.1%)	0.607
**Symptom score**			
decreased passage of stools, median (IQR)	0 (1)	0 (0)	0.097
hard stools, median (IQR)	0 (1)	0 (1)	0.705
nausea and vomiting, median (IQR)	0 (0)	0 (0)	0.270
acid regurgitation, median (IQR)	1 (1)	0.5 (1)	0.931
heartburn, median (IQR)	0 (1)	0 (1)	0.664
sucking sensations in the epigastrium, median (IQR)	0 (1)	0 (0)	1.000
abdominal pains, median (IQR)	0 (1)	0 (1)	0.737
abdominal distension, median (IQR)	2 (2)	1 (2)	0.002*
eructation, median (IQR)	0 (2)	0 (1)	0.236
loose stools, median (IQR)	0 (0)	0 (0)	0.710
increased flatus, median (IQR)	1 (3)	1 (3)	0.852
borborygmus, median (IQR)	0 (2)	0.5 (1)	0.806
increased passage of stools, median (IQR)	0 (0)	0 (0)	0.270
urgent need for defecation, median (IQR)	0 (1)	0 (2)	0.738
feeling of incomplete evacuation, median (IQR)	2 (2)	0 (1)	0.012*
Total score, median (IQR)	11 (12)	7.5 (9)	0.120

^a^Mann–Whitney U test was used to compare age, BMI and symptom score between SIBO + and SIBO− subjects; Fisher’s exact test was used to compare gender distribution and history of abdominal operation between SIBO + and SIBO− subjects. Bold values followed by * means P < 0.05.

### Overall Microbial Diversity and Composition of the Four Types of Samples of All Subjects

**FIGURE 1:**
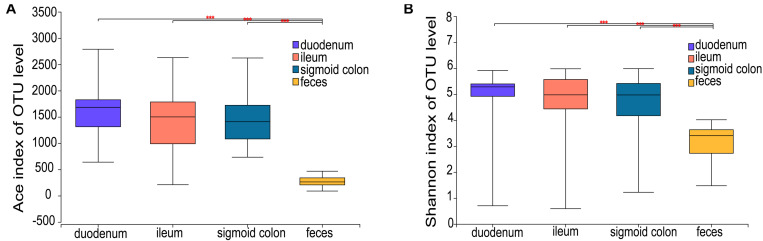
Microbial richness and diversity of the mucosal and fecal microbiota of all subjects. **(A,B)** Ace richness and Shannon diversity indices of mucosal and fecal samples: the richness and diversity were significantly lower in the fecal samples compared to the mucosal samples of the duodenum, ileum, and sigmoid colon. Data are presented as mean ± standard deviation of the mean for each sample type. The differences between groups were calculated using the Wilcoxon rank-sum test. ***P < 0.001.

**FIGURE 2:**
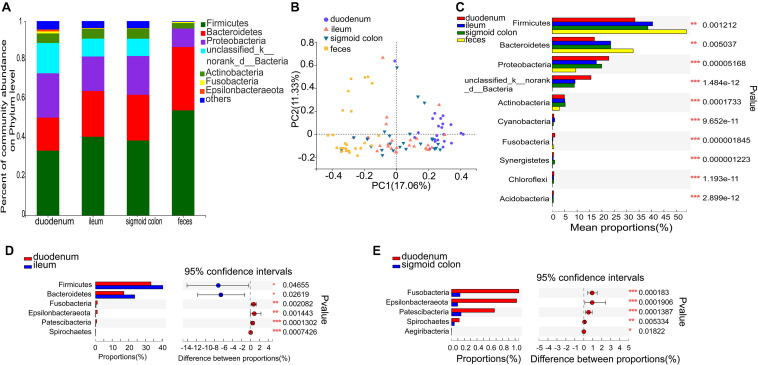
Distinct bacterial composition at the phylum level in the mucosal and fecal samples of all subjects. **(A)** The predominant phylum in the 4 types of samples. **(B)** PCoA analysis based on the bray_curtis distance showed that the bacterial composition of the fecal and duodenal mucosal samples were significantly different from the other two groups (ANOSIM *R* = 0.305, *P* = 0.001). **(C)** Firmicutes and Bacteroidetes were significantly enriched (*P* = 1.2 × 10^–3^ and *P* = 5 × 10^–3^, respectively), while Proteobacteria, unclassified_k_norank_d_Bacteria, and Actinobacteria were significantly lower in the fecal samples compared to the mucosal samples (*P* = 5.2 × 10^–5^, *P* = 1.5 × 10^–12^, and *P* = 1.7 × 10^–4^, respectively). **(D)** Firmicutes and Bacteroidetes were significantly lower in the duodenal mucosal samples than in the ileac mucosal samples (*P* = 0.047 and *P* = 0.026, relatively). **(E)** Fusobacteria, Epsilonbacteraeota, Patescibacteria, and Spirochetes were significantly enriched in the mucosal samples of the duodenum than in the mucosal samples of the sigmoid colon (*P* = 1.83 × 10^−4^, *P* = 1.9 × 10^−4^, *P* = 1.39 × 10^−4^, and *P* = 5.33 × 10^−3^, respectively). The phyla with < 0.1% abundance were aggregated as “others.” The Kruskal–Wallis H test was used to test differences among multiple groups with fdr controlling the false discovery rates. Wilcoxon rank-sum test was used to test differences between two groups. *P < 0.05, **P < 0.01, and ***P < 0.001.

The ACE richness index showed that the microbial richness values of the duodenum, ileum, and sigmoid colon were significantly higher than those of the feces (*P* < 0.001) (Figure 1A), and the Shannon diversity index indicated that the microbial diversities of the three gut mucosal sites were significantly higher than those of feces (*P* < 0.001) (Figure 1B). Similar results were obtained using the Chao and Sobs indices (Supplementary Figures 1A,B). Principal coordinates analysis (PCoA) based on the Bray-Curtis distance showed that the bacterial composition of either the fecal or duodenal mucosal samples was significantly different from those of the other three groups (ANOSIM *R* = 0.305, *P* = 0.001) (Figure 2B). The differences in microbial compositions among sample types and gut segments were further demonstrated by the partial least squares discriminant analysis (PLS-DA) (Supplementary Figure 1C). There were also no differences in alpha and beta diversities between the mucosal microbiota of the ileum and sigmoid colon (Supplementary Figure 1D).

### Bacterial Taxonomic Differences Among the Four Types of Samples of All Subjects

Species comprising more than 1% of the total microbiota were defined as predominant species. At the phylum level, the predominant phyla in the fecal microbiome were Firmicutes (54.06%), Bacteroidetes (32.59%), Proteobacteria (9.49%), and Actinobacteria (2.84%). The representative phyla were more diverse in the mucosal samples. The predominant phyla in the duodenal mucosal microbiome were Firmicutes (33.32%), Proteobacteria (22.79%), Bacteroidetes (17%), unclassified_k_norank_d_Bacteria (15.61%), Actinobacteria (4.91%), Fusobacteria (1.06%), and Epsilonbacteraeota (1.02%). The predominant phyla in the mucosal microbiome of the ileum and sigmoid colon were Firmicutes (40.45 and 38.54%, respectively), Bacteroidetes (23.55 and 23.54%, respectively), Proteobacteria (17.78 and 19.94%, respectively), unclassified_k_norank_d_Bacteria (9.18 and 8.9%, respectively), and Actinobacteria (5.07 and 5.23%, respectively) (Figure 2A and Supplementary Figures 2A–D). The mucosal microbiota in all three gut segments, especially in the duodenum, harbored more Proteobacteria, unclassified_k_norank_d_Bacteria, and Actinobacteria (*P* < 0.05), while those in the fecal samples harbored more Firmicutes and Bacteroidetes (*P* < 0.05) (Figure 2C). The mucosa of the duodenum showed lower relative proportions of Firmicutes and Bacteroidetes than the ileum (*P* < 0.05) (Figure 2D). It also showed higher relative proportions of Fusobacteria, Epsilonbacteraeota, Patescibacteria, and Spirochetes than the sigmoid colon (*P* < 0.01) (Figure 2E).

**FIGURE 3:**
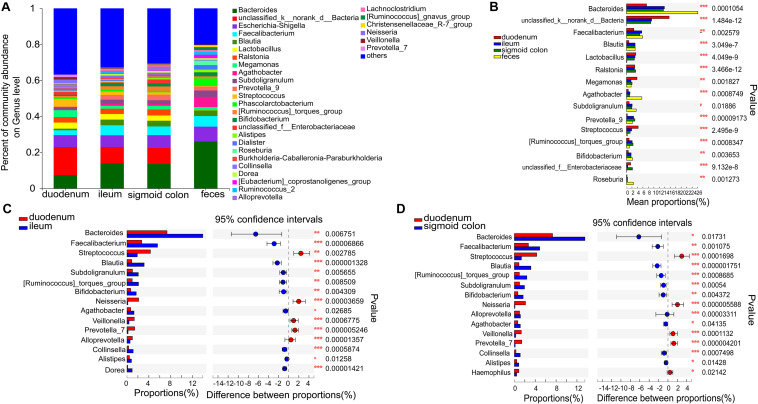
Distinct bacterial composition at the genus level in the mucosal and fecal samples of all subjects. **(A)** The predominant genera in the 4 types of samples. **(B)** Bacteroides (*P* < 0.001), Agathobacter (*P* < 0.001), and Roseburia (*P* < 0.01) were more abundant in the fecal samples compared to the mucosal samples. On the other hand, the relative numbers of unclassified_k_norank_d_bacteria (*P* < 0.001), lactobacillus (*P* < 0.001), Ralstonia (*P* < 0.001), and Streptococcus (*P* < 0.001) were more enriched in the mucosa compared to those in the feces. **(C,D)** Bacteroides (*P* < 0.05), Faecalibacterium (*P* < 0.01), Blautia (*P* < 0.001), Subdoligranulum (*P* < 0.01), Bifidobacterium (*P* < 0.01), and Agathobacter (*P* < 0.05) were relatively less abundant, whereas Streptococcus (*P* < 0.01), Neisseria (*P* < 0.001), Veillonella (*P* < 0.001), and Prevotella_7 (*P* < 0.001) were more abundant in the mucosa of the duodenum than in the ileum and sigmoid colon. The genera with < 0.1% abundance were aggregated as “others.” Kruskal–Wallis H test was used to test differences among multiple groups with fdr controlling the false discovery rates. Wilcoxon rank-sum test was used to test differences between two groups. *P < 0.05, **P < 0.01, and ***P < 0.001.

The six genera in the mucosa of the duodenum with the highest average proportions were unclassified_k_norank_d_bacteria (15.61%), Bacteroides (7.33%), Escherichia-Shigella (6.65%), Streptococcus (4.28%), Megamonas (3.86%), and Lactobacilli (3.41%); in the mucosa of the ileum were Bacteroides (13.87%), unclassified_k_norank_d_bacteria (9.18%), Escherichia-Shigella (6.42%), Faecalibacterium (5.58%), Blautia (3.14%), and Lactobacilli (3.12%); in the mucosa of sigmoid colon were Bacteroides (13.52%), unclassified_k_norank_d_bacteria (8.9%), Escherichia-Shigella (7.12%), Faecalibacterium (4.86%), Ralstonia (3.36%), and Blautia (3.17%); and in the feces were Bacteroides (26.06%), Escherichia-Shigella (8.21%), Faecalibacterium (5.9%), Agathobacter (5.64%), Phascolarctobacterium (4.27%), and Subdoligranulum (3.55%) (Figure 3A and Supplementary Figures 3A–D).

Bacteroides (*P* < 0.001), Agathobacter (*P* < 0.001), and Roseburia (*P* < 0.01) were more abundant in the fecal samples than in the mucosal samples (Figure 3B). On the other hand, the relative numbers of unclassified_k_norank_d_bacteria (*P* < 0.001), Lactobacillus (*P* < 0.001), Ralstonia (*P* < 0.001), and Streptococcus (*P* < 0.001) were higher in the mucosa than in the feces (Figure 3B). Compared to the ileum and sigmoid colon, Bacteroides (*P* < 0.05), Faecalibacterium (*P* < 0.01), Blautia (*P* < 0.001), Subdoligranulum (*P* < 0.01), [Ruminococcus]_torques_group (*P* < 0.01), Bifidobacterium (*P* < 0.01), and Agathobacter (*P* < 0.05) were relatively less abundant, whereas Streptococcus (*P* < 0.01), Neisseria (*P* < 0.001), Veillonella (*P* < 0.001), and Prevotella_7 (*P* < 0.001) were more abundant in the duodenal mucosa (Figures 3C,D). Although there was a high similarity between the bacterial species in the mucosa of the ileum and sigmoid colon, there were some genera with significantly different proportions. [Eubacterium]_ventriosum_group (*P* < 0.05) and norank_f__TRA3-20 (*P* < 0.05) were significantly more abundant in the mucosa of the ileum, while Anaerococcus (*P* < 0.05), Ruminiclostridium_1 (*P* < 0.05), Roseomonas (*P* < 0.05), Norank_f_veillonellacece (*P* < 0.01), and Herbaspirillum (*P* < 0.05) were more abundant in the mucosa of the sigmoid colon (Supplementary Figure 1E).

### Mucosa-Associated Bacterial Microbiota of Multiple Gut Segments, but Not Fecal Microbiota, Were Significantly Different Between SIBO + and SIBO− Subjects

**FIGURE 4:**
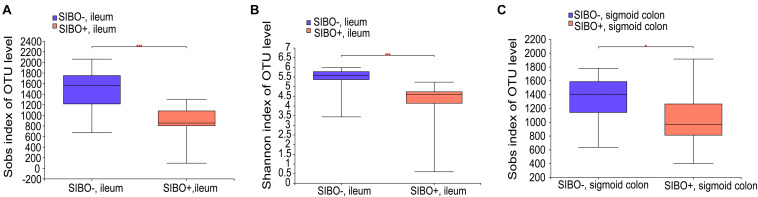
Microbial richness and diversity of the mucosa-associated microbial community in the SIBO + and SIBO− subjects. **(A,B)** The Sobs richness and Shannon diversity indices of the ileac mucosal samples were significantly lower in the SIBO + subjects compared to the SIBO− subjects. **(C)** The Sobs richness index of the mucosal samples of the sigmoid colon was significantly lower in the SIBO + subjects than in the SIBO− subjects. *P < 0.05 and ***P < 0.001.

**FIGURE 5:**
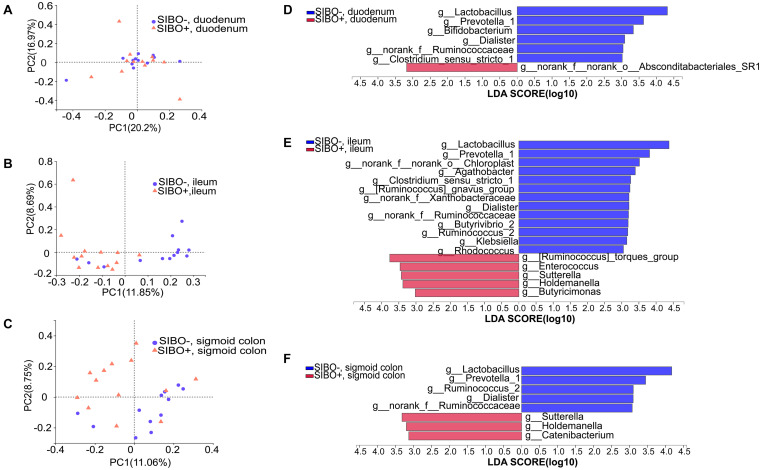
Distinct bacterial composition in the mucosal samples of the SIBO + and SIBO− groups. **(A–C)** PCoA analysis. The bacterial community compositions were clustered significantly separately between the SIBO + and SIBO− groups in the duodenal mucosa based on abund_jaccard distance (ANOSIM *R* = 0.102, *P* = 0.039), in the ileac mucosa based on binary_jaccard (ANOSIM *R* = 0.216, *P* = 0.002), and in the mucosa of sigmoid colon based on binary_jaccard (ANOSIM *R* = 0.155, *P* = 0.007). **(D–F)** Linear discriminant analysis (LDA) identified distinct bacterial genera that were enriched in the SIBO + and SIBO− groups. Genera with LDA score > 3 were considered significant. The abundances of 7, 18, and 8 genera differed significantly in the mucosal samples of duodenum, ileum, and sigmoid colon between the SIBO + and SIBO− groups.

Compared with the SIBO− group, the microbiota richness values based on the Sobs index of the mucosa of the ileum and sigmoid colon were significantly decreased (*P* < 0.001 and *P* < 0.05, respectively) (Figures 4A,C) and the microbiota diversity based on the Shannon index of the ileum mucosa was significantly reduced (*P* < 0.001) (Figure 4B) in the SIBO + group. No significant differences in the microbial diversity were observed in neither duodenum mucosa nor feces between the SIBO + and SIBO− groups (Supplementary Figures 4A,B). Clustering in the bacterial community was significantly different between the SIBO + and SIBO− groups in the duodenal mucosa based on abund_jaccard distance (*P* = 0.039) (Figure 5A), in the ileac mucosa based on binary_jaccard (*P* = 0.002) (Figure 5B), and in the mucosa of the sigmoid colon based on binary_jaccard (*P* = 0.007) (Figure 5C). However, this finding was not observed in the fecal samples (Supplementary Figure 4C). Overall, the mucosal microbiota of the duodenum, ileum, and sigmoid colon were structurally different in the SIBO + subjects compared to SIBO− subjects.

### Taxonomic Differences in Mucosal Microbiota of SIBO + and SIBO− Subjects

Differential expression analysis showed that the relative abundances of multiple genera in the duodenum, ileum, and sigmoid colon mucosa were significantly different between the SIBO + and SIBO− groups. LEfSe (LDA score > 3) was used to detect the microbial species that most likely explains the differences between the SIBO− and SIBO + groups. In the duodenal mucosa, six genera were significantly enriched in the SIBO− group compared to the SIBO + group: Lactobacillus, Prevotella_1, Bifidobacterium, Dialister, norank_f__Ruminococcaceae, and Clostridium_sensu_stricto_1. Additionally, only the genus norank_f__norank_o__Absconditabacteriales_SR1 was enriched in the SIBO + group (Figure 5D). In the ileac mucosa, 13 genera were more abundant in the SIBO− group: Lactobacillus, Prevotella_1, norank_f__norank_o__Chloroplast, Agathobacter, Clostridium_sensu_stricto_1, [Ruminococcus] _gnavus_group, norank_f__Xanthobacteraceae, Dialister, norank_f__Ruminococcaceae, Butyrivibrio_2, Ruminococcus_2, Klebsiella, and Rhodococcus. However, five genera were more abundant in the SIBO + group: [Ruminococcus]_torques_group, Enterococcus, Sutterella, Holdemanella, and Butyricimonas (Figure 5E). In the sigmoid mucosa, five genera were more abundant in the SIBO− group: Lactobacillus, Prevotella_1, Ruminococcus_2, Dialister, and g__norank_f__Ruminococcaceae. However, three genera were more abundant in the SIBO + group: Sutterella, Holdemanella, and Catenibacterium (Figure 5F).

To further identify the shared genera involved in the pathophysiology of SIBO, we found five shared genera in the mucosal samples of the duodenum and ileum and seven shared genera in the mucosal samples of the ileum and sigmoid colon which were significantly different between the SIBO + and SIBO− subjects. Of these, four genera (Lactobacillus, Prevotella_1, Dialister, and norank_f__Ruminococcaceae) had similar changes among the duodenal, ileac, and sigmoid colonic samples in the SIBO + subjects compared to the SIBO− subjects.

### Identification of Microbial Signature of SIBO + Subjects

**FIGURE 6:**
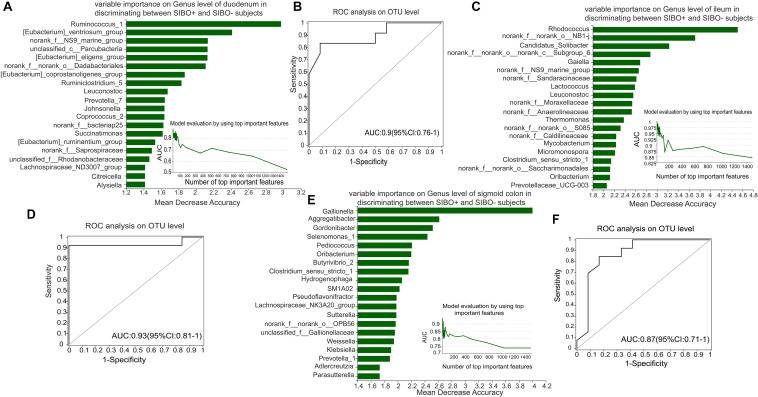
Identification of microbial signatures of SIBO + subjects. Random forest analysis was used to identify taxa in the mucosal samples of the duodenum, ileum, and sigmoid colon to distinguish the SIBO + and SIBO− subjects. The genera were ranked in descending order according to their importance to the accuracy of the model. The insert shows the AUC verification results. X represents the number of species in the importance ranking and Y represents the AUC value. According to the solid point with the largest Y value, the corresponding X value was considered the ultimate number of top species to build a prediction model. The prediction performance was assessed by ROC analysis. **(A,B)** The random forest analysis showed that use of the top 4 important features (Ruminococcus_1, [Eubacterium]_ventriosum_group, norank_f__NS9_marine_group, and unclassified_c__Parcubacteria) of the duodenal mucosal samples for prediction of SIBO was the most optimal model, and ROC Analysis assessed its prediction performance with an AUC value of 0.9 (95% CI: 0.76–1). **(C,D)** In the ileum, random forest analysis found that use of the top 3 important features (Rhodococcus, norank_f__norank_o__NB1-j, and Candidatus_Solibacter) to construct the prediction model yielded an AUC value of 0.93 (95% CI: 0.81–1) based on ROC analysis. **(E,F)** In the sigmoid colon, the random forest analysis found that use of the top 6 important features (Gallionella, Aggregatibacter, Gordonibacter, Selenomonas_1, Pediococcus, and Oribacterium) to build the prediction model yielded an AUC value of 0.87 (95% CI: 0.71–1) based on ROC analysis.

To identify a characteristic microbiome with the potential to distinguish between SIBO− and SIBO + patients, a random forest analysis was done to screen for the most important biomarker. In the duodenum, the random forest analysis showed that the top 4 important features (Ruminococcus_1, [Eubacterium]_ventriosum_group, norank _f__NS9_marine_group, and unclassified_c__Parcubacteria) exhibited the highest predictive power. ROC analysis was then performed to evaluate the performance of the model, wherein an AUC value of 0.9 (95% CI: 0.76–1) was obtained (Figures 6A,B). In the ileum, the random forest analysis yielded the top 3 important features (Rhodococcus, norank_f__norank_o__NB1-j, and Candidatus_Solibacter) that were used to construct the prediction model, in which ROC analysis showed an AUC value of 0.93 (95% CI: 0.81–1) (Figures 6C,D). In the sigmoid colon, the random forest analysis showed the top 6 important features (Gallionella, Aggregatibacter, Gordonibacter, Selenomonas_1, Pediococcus, and Oribacterium) which were used to build a prediction model with an AUC value of 0.87 (95% CI: 0.71–1) based on ROC analysis (Figures 6E,F).

## Discussion

Although SIBO has been explored for decades, previous studies have mainly focused on investigating aspiration cultures, which are largely inadequate for the characterization of the complex bacterial community structure of the gut in patients with SIBO (Shin et al., 2019; Yang et al., 2020). In addition, studies have found that the mucosal niche and luminal niche are two different ecosystems (Ringel et al., 2015; Dreskin et al., 2021). Thus, we sought to characterize the microbial profiles of the gastrointestinal tract mucosa and feces to provide a more comprehensive picture of the gut microbiota in patients with SIBO through 16S rRNA gene sequencing.

We compared the diversities and compositions of mucosa-associated and fecal microbiota. Through this, we found that the alpha-diversity of fecal microbiota was significantly lower than those of mucosa-associated microbiota, indicating that fecal microbiota is clearly separate from mucosa-associated microbiota. Due to the limited research on the sequencing of the microbiota in patients with SIBO at present, it is difficult to find a matching study to compare with our study. A few studies have compared fecal flora to mucosal flora, all of which were studies of other diseases. A prior study by Rangel et al. (2015) observed that while there were no differences in microbial diversities between fecal and unprepared sigmoid colon mucosal samples in healthy controls, lower diversity was observed in fecal microbiota than in mucosal microbiota in patients with IBS. However, Ringel et al. (2015) also observed higher alpha-diversity in fecal samples compared to the unprepared distal colon mucosal samples in healthy individuals. Due to differences in the study population and sampling types, these results could not be fairly compared with those of our study.

Our study supports the notion that mucosal bacteria and luminal bacteria are two different ecosystems with different physiological functions (Carroll et al., 2011). The dominant phyla and genera were different in the fecal microbiota compared to the mucosal microbiota. While luminal microbiota are mainly engaged in the digestive process through indirect interaction with the host through metabolite and toxin production, mucosal microbiota are of particular concern because they generally interact more directly with the host (Carroll et al., 2011; Dlugosz et al., 2015; Maharshak et al., 2018). We also found that the mucosal bacterial composition of the duodenum was significantly different from those of the ileum and sigmoid colon, suggesting that the composition of intestinal bacteria is highly affected by the type of sample and gut site.

In our study, no differences were observed in the alpha-diversity or beta-diversity of the feces of the SIBO + subjects compared to those of the SIBO− subjects. In line with this, Yang et al. (2020) examined fecal microbiota in patients with IBS and found no significant difference in fecal microbial diversity and composition in patients with SIBO + IBS-D compared to patients with SIBO−IBS-D. Due to differences in the study population and sampling types, the results of Yang et al. could not be fairly compared with our study, and only a preliminary conclusion can be made that the findings in the fecal samples are partially consistent with our study.

While the duodenal microbial composition was observed to be distinctly different in the SIBO + subjects compared with the SIBO− subjects, this was not reflected in terms of alpha-diversity. Furthermore, the mucosal microbial compositions were significantly different and the microbial richness values were significantly reduced in both the ileum and sigmoid colon of the SIBO + subjects compared with the SIBO− subjects. Ours is the first study to characterize the ileac mucosal microbiota, showing that the microbial diversity and composition in the ileac mucosa was significantly different between the SIBO + and SIBO− subjects. This suggests that dysbiosis in the ileum may be an important feature of SIBO. Since there are limited studies on the ileac mucosal microbiota in SIBO + patients, it is difficult to find appropriate comparisons for our study. A previous study reported that the microbial diversities and compositions were distinctly different in patients with SIBO + IBS-D compared with patients with SIBO− IBS-D in the duodenal and rectal mucosa (Yang et al., 2020). Since the study population and sample types are not similar, these findings are only partially consistent with our conclusions. While Shin et al. (2019) reported significantly lower alpha-diversity in the jejunal mucosa of patients with SIBO diagnosed by culture compared to patients without SIBO, they did not collect samples of the duodenum, ileum, and sigmoid colon. Leite et al. (2020) sequenced the 16S rRNA of duodenal respirates and showed decreased microbial diversity in patients with SIBO compared to patients without SIBO. However, comparison of this study with our findings is challenging due to inconsistencies in sample types and intestinal segments. On the other hand, these findings confirm that the intestinal microbiota community is region-specific (Hillman et al., 2017; Sundin et al., 2020), indicating that collection of multiple samples along varying sites of the gastrointestinal tract is needed to explore the spatial heterogeneity of the gut microbiome.

We also found that the relative abundances of multiple taxa were different in the mucosa of the duodenum, ileum, and sigmoid colon between the SIBO + and SIBO− subjects. Interestingly, some of the shifted genera showed consistent changes across the gut segments. Four genera (Lactobacillus, Prevotella_1, Dialister, and norank_f__Ruminococcaceae) were decreased in the mucosa of the duodenum, ileum, and sigmoid colon in the SIBO + subjects compared to the SIBO− subjects. Lactobacillus is recognized as probiotics because of its beneficial effects on the host (Zhang et al., 2018). Probiotics such as Lactobacillus maintain and promote microbial homeostasis as well as inhibit the invasion and colonization of pathogens. Untargeted microbiome studies have supported the profound impact of intestinal Lactobacillus on human health (Heeney et al., 2018). A systemic review of 31 studies has shown that the relative abundant of Lactobacillus and some butyrogenic genera were reduced in patients with colorectal cancer (Borges-Canha et al., 2015). Furthermore, a meta-analysis concluded that the relative abundant of Lactobacillus was decreased in patients with diarrhea-dominant IBS compared to healthy subjects (Liu et al., 2017). Another recently published meta-analysis showed that Lactobacillus improved clinical symptoms in patients with ulcerative colitis (Ganji-Arjenaki and Rafieian-Kopaei, 2018). On the other hand, a study by Jiang et al. (2018) showed that Dialister was found to be decreased in children with attention-deficit/hyperactivity disorder compared to healthy controls. A prior study also found that the relative abundance of Dialister in children with autism spectrum disorder was decreased (Andreo-Martínez et al., 2020). Additionally, Prevotella, which is a short-chain fatty acid producing anaerobes (Mocanu et al., 2021), was found to be implicated in the development of severe gastrointestinal symptoms (Tan et al., 2021). These bacteria with the same changing trend in different intestine sites may guide future research on understanding the pathophysiology behind SIBO or provide insight into potential therapeutic targets.

Random forest analysis was used to rank species in the mucosal microbiota of the three intestinal segments based on importance. We found that the top four important genera in the duodenum, the top three important genera in the ileum, and the top six important genera in the sigmoid colon exhibited good predictive efficacy for SIBO, with AUC values of 0.9, 0.93, and 0.87, respectively. This implies that the identified microbial signatures may be a potential tool for SIBO prediction.

Although our study attempted to provide a comprehensive understanding of the microbiota in the mucosa of the upper (duodenum), middle (ileum), and lower (sigmoid colon) gastrointestinal tracts as well as feces of patients with SIBO, there were some limitations that need to be addressed in future studies. First, we did not establish a validation cohort to further test the discriminative ability of the prediction model since the sample size was not large enough. Second, samples of the ileum and sigmoid colon were collected after intestinal preparation, which may have influenced the mucosal flora. However, this could not be avoided in our study since the mucosa of the middle and lower GI, especially the ileac mucosal specimens, cannot be obtained without intestinal preparation. Finally, it is important to note that a comprehensive study of the microbiome must include studies on viruses, fungi, and archaea.

Overall, we characterized the mucosal microbiota in the gastrointestinal tract and feces of SIBO + subjects diagnosed by LBT, and identified some compositional dysbiosis compared to SIBO− subjects. These findings may guide future investigation on the pathogenesis of SIBO as well as potential therapeutic targets. Additionally, we identified mucosal microbial signatures in SIBO + subjects that require further validation in larger cohorts.

## Data Availability Statement

The datasets presented in this study can be found in online repositories. The names of the repository/repositories and accession number(s) can be found below: https://www.ncbi.nlm.nih.gov/, PRJNA728515.

## Ethics Statement

The studies involving human participants were reviewed and approved by the Institutional Ethical Review Committee of the Chinese PLA General Hospital. The patients/participants provided their written informed consent to participate in this study.

## Author Contributions

JW, RZ, and JL designed the study. JW obtained funding and revised the manuscript. JW, JM, YuL, and ST conducted the study and collected the samples. JL and YiL performed the bioinformatics and statistical analysis, and interpreted the data. JL wrote the manuscript. All authors reviewed the manuscript and approved the final version.

## Conflict of Interest

The authors declare that the research was conducted in the absence of any commercial or financial relationships that could be construed as a potential conflict of interest.

## Publisher’s Note

All claims expressed in this article are solely those of the authors and do not necessarily represent those of their affiliated organizations, or those of the publisher, the editors and the reviewers. Any product that may be evaluated in this article, or claim that may be made by its manufacturer, is not guaranteed or endorsed by the publisher.
