# Supplementary material for: Mucosa-Associated Microbial Profile Is Altered in Small Intestinal Bacterial Overgrowth
Source: Front Microbiol. 2021 Jul 30;12:710940. doi: 10.3389/fmicb.2021.710940 (PMC8372370; doi:10.3389/fmicb.2021.710940)
Supplement: Supplementary file 1 [file Data_Sheet_1.docx]

Supplementary Material

# Supplementary Figures

## Supplementary Figure 1


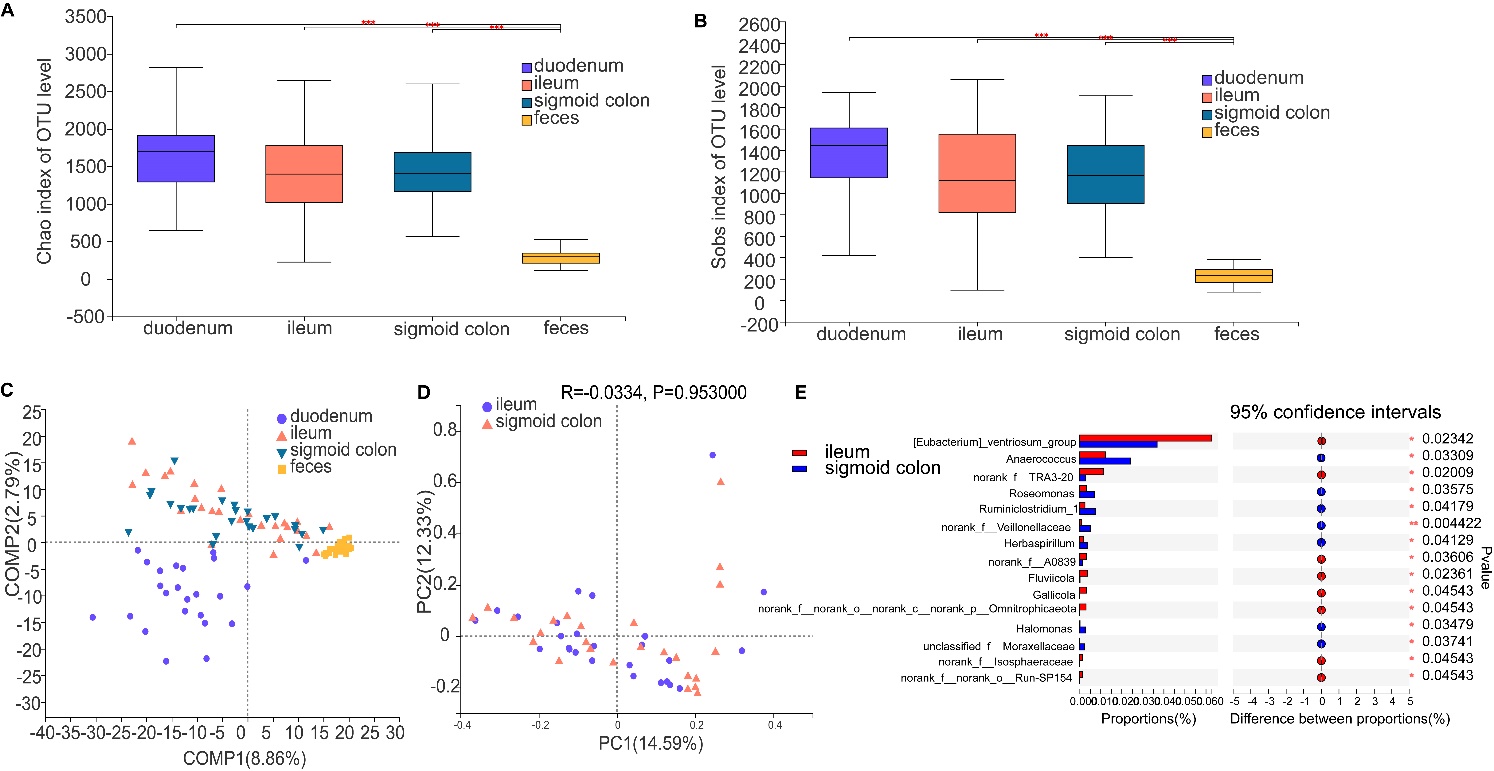


**Supplementary Figure 1.** Microbial diversity and composition of the four types of samples. (A, B) Chao richness and Sobs richness indices of mucosal and fecal samples: the richness was significantly lower in the fecal samples compared to the mucosal samples of the duodenum, ileum, and sigmoid colon. Data are presented as mean ± standard deviation of the mean for each sample type. The differences between groups were calculated using the Wilcoxon rank-sum test. (C) Partial least squares discriminant analysis (PLS-DA) showed that the bacterial composition of either the fecal or duodenal mucosal samples was significantly different from the other groups. (D) PCoA analysis based on the bray_curtis distance showed no differences in beta diversities between the mucosal microbiota of the ileum and sigmoid colon (ANOSIM R=0.0334, *P=*0.953). (E) Analysis of the differences between two groups showed that [Eubacterium]_ventriosum_group (*P<*0.05) and norank_f__TRA3-20 (*P<*0.05) were significantly more abundant in the mucosa of the ileum, while Anaerococcus (*P<*0.05) , Ruminiclostridium_1 (*P<*0.05), Roseomonas (*P<*0.05), Norank_f_veillonellacece (*P<*0.01), and Herbaspirillum (*P<*0.05) were more abundant in the mucosa of the sigmoid colon. **P<*0.05, ***P<*0.01, ****P<*0.001.

## Supplementary Figure 2


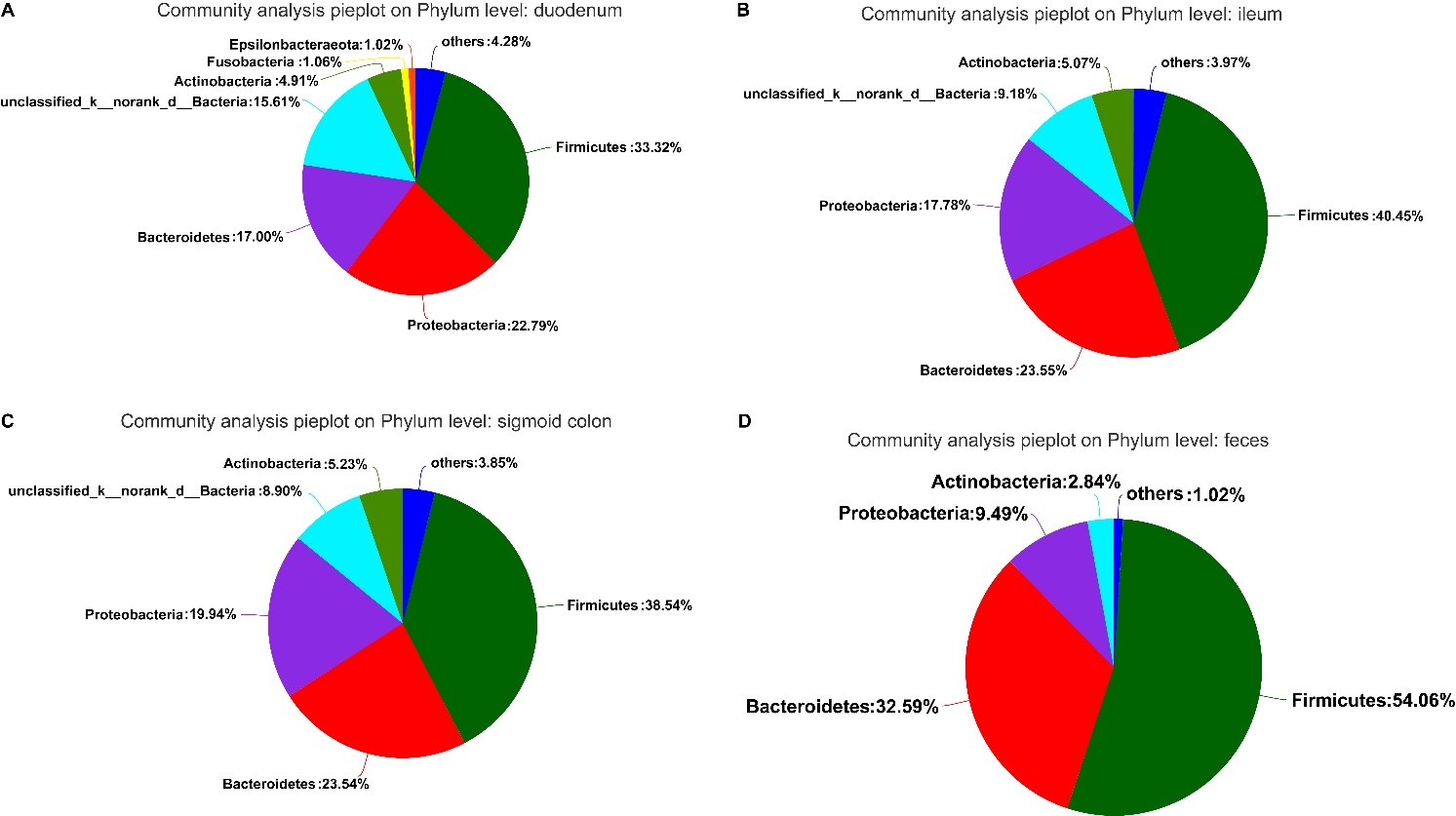


**Supplementary Figure 2.** Microbial community of the four types of samples at phylum level. Species comprising more than 1% of the total microbiota were defined as predominant species. (A) The predominant phyla in the duodenal mucosal microbiome were Firmicutes (33.32%), Proteobacteria (22.79%), Bacteroidetes (17%), unclassified_k_norank_d_Bacteria (15.61%), Actinobacteria (4.91%), Fusobacteria (1.06%), and Epsilonbacteraeota (1.02%). (B, C) The predominant phyla in the mucosal microbiome of the ileum and sigmoid colon were Firmicutes (40.45% and 38.54%, respectively), Bacteroidetes (23.55% and 23.54%, respectively), Proteobacteria (17.78% and 19.94%, respectively), unclassified_k_norank_d_Bacteria (9.18% and 8.9%, respectively), and Actinobacteria (5.07% and 5.23%, respectively). (D) The predominant phyla in the fecal microbiome were Firmicutes (54.06%), Bacteroidetes (32.59%), Proteobacteria (9.49%), and Actinobacteria (2.84%).

## Supplementary Figure 3


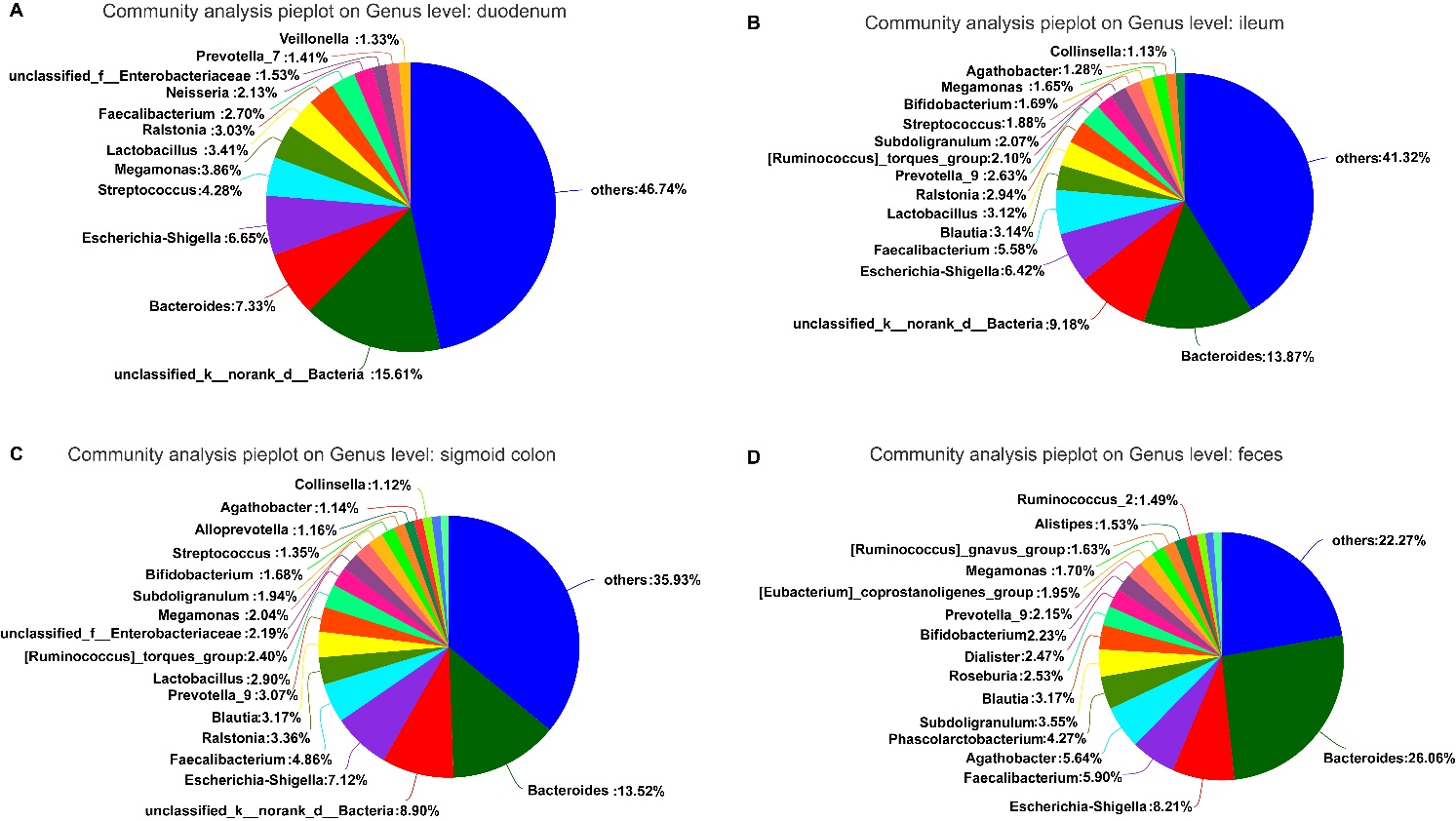


**Supplementary Figure 3.** Microbial community of the four types of samples at genus level. (A) The six genera in the duodenal mucosa with the highest average proportions were unclassified_k_norank_d_bacteria (15.61%), Bacteroides (7.33%), Escherichia-Shigella (6.65%), Streptococcus (4.28%), Megamonas (3.86%), and Lactobacilli (3.41%). (B) The six genera in the ileac mucosa with the highest average proportions were Bacteroides (13.87%), unclassified_k_norank_d_bacteria (9.18%), Escherichia-Shigella (6.42%), Faecalibacterium (5.58%), Blautia (3.14%), and Lactobacilli (3.12%). (C) The six genera in the mucosa of the sigmoid colon with the highest average proportions were Bacteroides (13.52%), unclassified_k_norank_d_bacteria (8.9%), Escherichia-Shigella (7.12%), Faecalibacterium (4.86%), Ralstonia (3.36%), and Blautia (3.17%). (D) The six genera in the feces with the highest average proportions were Bacteroides (26.06%), Escherichia-Shigella (8.21%), Faecalibacterium (5.9%), Agathobacter (5.64%), Phascolarctobacterium(4.27%), and Subdoligranulum (3.55%).

## Supplementary Figure 4


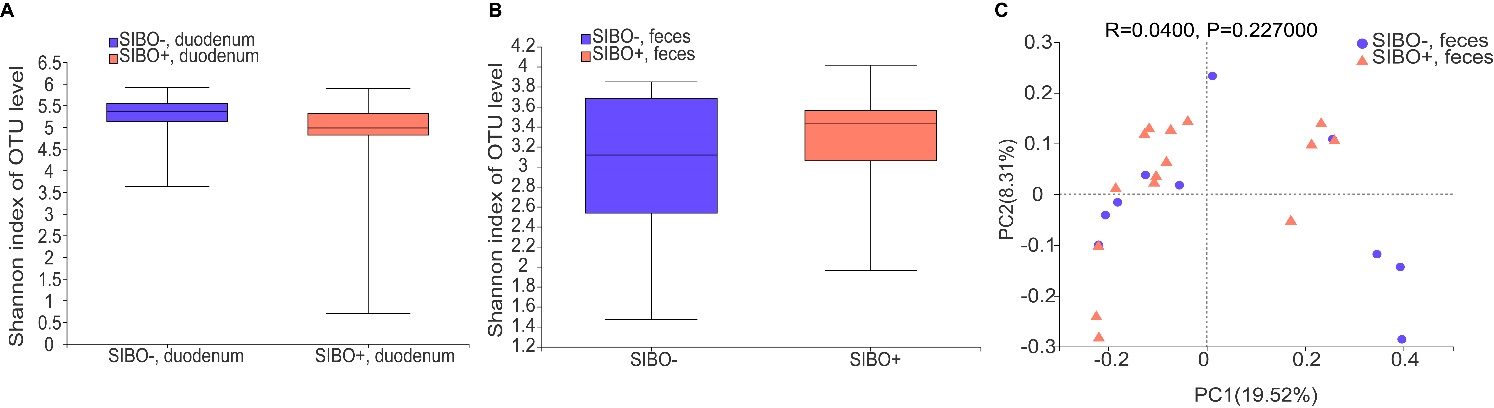


**Supplementary Figure 4.** Microbial richness and diversity of the mucosa-associated and fecal microbial community in the SIBO+ and SIBO- subjects. (A, B) The Shannon diversity indices of the duodenal mucosal and fecal samples showed no differences between the SIBO+ and SIBO- subjects. (C) PCoA analysis based on binary_jaccard showed the bacterial community compositions were clustered together between the SIBO+ and SIBO- groups in the feces (ANOSIM R=0.04, *P=*0.227).
